# ASD-DiagNet: A Hybrid Learning Approach for Detection of Autism Spectrum Disorder Using fMRI Data

**DOI:** 10.3389/fninf.2019.00070

**Published:** 2019-11-27

**Authors:** Taban Eslami, Vahid Mirjalili, Alvis Fong, Angela R. Laird, Fahad Saeed

**Affiliations:** ^1^Department of Computer Science, Western Michigan University, Kalamazoo, MI, United States; ^2^School of Computing and Information Science, Florida International University, Miami, FL, United States; ^3^Department of Computer Science and Engineering, Michigan State University, East Lansing, MI, United States; ^4^Department of Physics, Florida International University, Miami, FL, United States

**Keywords:** fMRI, ASD, SLP, autoencoder, ABIDE, classification, data augmentation

## Abstract

Heterogeneous mental disorders such as Autism Spectrum Disorder (ASD) are notoriously difficult to diagnose, especially in children. The current psychiatric diagnostic process is based purely on the behavioral observation of symptomology (DSM-5/ICD-10) and may be prone to misdiagnosis. In order to move the field toward more quantitative diagnosis, we need advanced and scalable machine learning infrastructure that will allow us to identify reliable biomarkers of mental health disorders. In this paper, we propose a framework called ASD-DiagNet for classifying subjects with ASD from healthy subjects by using *only* fMRI data. We designed and implemented a joint learning procedure using an autoencoder and a single layer perceptron (SLP) which results in improved quality of extracted features and optimized parameters for the model. Further, we designed and implemented a data augmentation strategy, based on linear interpolation on available feature vectors, that allows us to produce synthetic datasets needed for training of machine learning models. The proposed approach is evaluated on a public dataset provided by Autism Brain Imaging Data Exchange including 1, 035 subjects coming from 17 different brain imaging centers. Our machine learning model outperforms other state of the art methods from 10 imaging centers with increase in classification accuracy up to 28% with maximum accuracy of 82%. The machine learning technique presented in this paper, in addition to yielding better quality, gives enormous advantages in terms of execution time (40 min vs. 7 h on other methods). The implemented code is available as GPL license on GitHub portal of our lab (https://github.com/pcdslab/ASD-DiagNet).

## 1. Introduction

Mental disorders such as Autism Spectrum Disorder (ASD) are heterogeneous disorders that are notoriously difficult to diagnose, especially in children. The current psychiatric diagnostic process is based purely on behavioral observation of symptomology (DSM-5/ICD-10) and may be prone to misdiagnosis (Nickel and Huang-Storms, 2017). There is no quantitative test that can be prescribed to patients that may lead to definite diagnosis of a person. Such quantitative and definitive tests are a regular practice for other diseases such as diabetes, HIV, and hepatitis-C. It is widely known that defining and diagnosing mental health disorders is a difficult process due to overlapping nature of symptoms, and lack of a biological test that can serve as a definite and quantified gold standard (National Collaborating Centre for Mental Health (UK), 2018). ASD is a lifelong neuro-developmental brain disorder which causes social impairments like repetitive behavior and communication problems in children. More than 1% of children suffer from this disorder and detecting it at early ages can be beneficial. Studies show that some demographic attributes like gender and race vary among ASD and healthy individuals such that males are four times more prone to ASD than females (Baio et al., 2018). Diagnosing ASD has been explored from different aspects, like monitoring behavior, extracting discriminatory patterns from the demographic information and analyzing the brain data. Behavioral data such as eye movement and facial expression are studied in Liu et al. (2016), Jaiswal et al. (2017), Zunino et al. (2018). For instance, Zunino et al. classified ASD from healthy subjects by applying recurrent neural network to the video clips recorded from them (Zunino et al., 2018).

Quantitative analysis of brain imaging data can provide valuable biomarkers that result in more accurate diagnosis of brain diseases. Machine learning techniques using brain imaging data [e.g., Magnetic Resonance Imaging (MRI) and functional Magnetic Resonance Imaging (fMRI)] have been extensively used by researchers for diagnosing brain disorders like Alzheimer's, ADHD, MCI, and Autism (Colby et al., 2012; Peng et al., 2013; Yang et al., 2014; Deshpande et al., 2015; Hosseini-Asl et al., 2016; Khazaee et al., 2017; Eslami and Saeed, 2018b, 2019).

In this paper, we focus on classifying subjects suffering from ASD from healthy control subjects using fMRI data. We propose a method called *ASD-DiagNet* which consists of an autoencoder and a SLP. These networks are used for extracting lower dimensional features in a hybrid manner and the trained perceptron is used for the final round of classification. In order to enlarge the size of the training set, we designed a data augmentation technique which generates new data in feature space by using available data in the training set.

Detecting ASD using fMRI data has recently gained a lot of attention, thanks to Autism Brain Imaging Data Exchange (ABIDE) initiative for providing functional and structural brain imaging datasets collected from several brain imaging centers around the world (Craddock et al., 2013). Many studies and methods have been developed based on ABIDE data (Iidaka, 2015; Chen et al., 2016; Abraham et al., 2017; Heinsfeld et al., 2018; Itani and Thanou, 2019). Some studies included a subset of this dataset based on specific demographic information to analyze their proposed method. For example, Iidaka (2015) used probabilistic neural network for classifying resting state fMRI (rs-fMRI) data of subjects under 20 years old. In another work, Plitt et al. (2015) used two sets of rs-fMRI data, one containing 118 male individuals (59 ASD; 59 TD) and the other containing 178 age and IQ matched individuals (89 ASD; 89 TD) from ABIDE dataset and achieved 76.67% accuracy. Besides using fMRI data, some studies also included structural and demographic information of subjects for diagnosing ASD. For example, Parisot et al. (2018) proposed a framework based on Graph Convolutional Networks that achieved 70.4% accuracy. In their work, they represented the population as a graph in which nodes are defined based on imaging features and phenotypic information describe the edge weights. In another study, Sen et al. (2018) proposed a new algorithm which combines structural and functional features from MRI and fMRI data and got 64.3% accuracy by using 1111 total healthy and ASD subjects. Nielsen et al. (2013) obtained 60% accuracy on a group of 964 healthy and ASD subjects using the functional connectivity between 7266 regions and demographic information like age, gender, and handedness attributes. In another study, Parikh et al. (2019) tested the performance of different machine learning methods on demographic information provided by ABIDE dataset including age, gender, handedness, and three individual measures of IQ.

Machine learning techniques such as Support Vector Machines (SVM) and random forests are explored in multiple studies (Abraham et al., 2017; Subbaraju et al., 2017; Bi et al., 2018b; Fredo et al., 2018). For instance, Chen et al. (2016) investigated the effect of different frequency bands for constructing brain functional network, and obtained 79.17% accuracy using SVM technique applied to 112 ASD and 128 healthy control subjects.

Recently, using neural networks and deep learning methods such as autoencoders, Deep Neural Network (DNN), Long Short Term Memory (LSTM), and Convolutional Neural Network (CNN) have also become very popular for diagnosing ASD (Dvornek et al., 2017; Guo et al., 2017; Bi et al., 2018a; Brown et al., 2018; Khosla et al., 2018; Li et al., 2018). Brown et al. (2018) obtained 68.7% classification accuracy on 1, 013 subjects composed of 539 healthy control and 474 with ASD, by proposing an element-wise layer for DNNs which incorporated the data-driven structural priors.

Most recently, Heinsfeld et al. (2018) used a deep learning based approach and achieved 70% accuracy for classifying 1, 035 subjects (505 ASD and 530 controls). They claimed this approach improved the state of the art technique. In their technique, distinct pairwise Pearson's correlation coefficients were considered as features. Two stacked denoising autoencoders were first pre-trained in order to extract lower dimensional data. After training autoencoders, their weights were applied to a multi-layer perceptron classifier (fine-tuning process) which was used for the final classification. However, they also performed classification for each of the 17 sites included in ABIDE dataset separately, and the average accuracy is reported as 52%. The low performance on individual sites was justified to be due to the lack of enough training samples for intra-site training.

Generally, most related studies for ASD diagnosis using machine learning techniques have only considered a subset of ABIDE dataset, or they have incorporated other information besides fMRI data in their model. There are few studies such as Heinsfeld et al. (2018), which only used fMRI data without any assumption on demographic information and analyzed *all* the 1, 035 subjects in ABIDE dataset. To the best of our knowledge (Heinsfeld et al., 2018) is currently state of the art technique for ASD diagnosis on whole ABIDE dataset, which we use as the baseline for evaluating our proposed method.

Although employing other types of information like anatomical features and demographic attributes of subjects could provide more knowledge to the model and may increase its accuracy, the goal of our study is to merely design a quantitative model for ASD diagnosis based on the functional data of the brain. This model can be used in conjunction with other tools assisting clinicians to diagnose ASD with more precision. Another aspect that we targeted in this study is the running time of the model. Unfortunately, the running time required for training the model or analyzing the data is not discussed in most of research papers mentioned above. Achieving high diagnosis accuracy in a shorter amount of time would be more desirable in clinical studies. Deep learning models are time consuming techniques due to the huge number of parameters that should be optimized. Although utilizing GPUs has reduced the running time needed for training the models tremendously, it still depends on the architecture of the model and size of the data. We considered the running time of the model as a factor while designing the architecture of our model. Using our hybrid learning strategy the model needs fewer number of iterations for training, which reduces the running time of the model. We also decreased the number of features by keeping anti-correlated and highly correlated functional connections and removing the rest, which reduces the size of the network significantly.

The structure of this paper is as follows: First, in section 2 we provide a brief introduction to fMRI data, the dataset we used in this study and explain ASD-DiagNet method in detail. In section 3, we describe the experiment setting and discuss the results of ASD-DiagNet. Finally, in section 4, we conclude the paper and discuss the future direction.

## 2. Materials and Methods

### 2.1. Functional Magnetic Resonance Imaging and ABIDE Dataset

Functional Magnetic Resonance Imaging (fMRI) is a brain imaging technique that is used for studying brain activities (Lindquist et al., 2008; Eslami and Saeed, 2018a). In fMRI data, the brain volume is represented by a group of small cubic elements called voxels. A time series is extracted from each voxel by keeping track of its activity over time. Scanning the brain using fMRI technology while the subject is resting is called resting state fMRI (rs-fMRI), which is widely used for analyzing brain disorders. In this study, we used preprocessed ABIDE-I dataset that is provided by the ABIDE initiative. This dataset consists of 1112 rs-fMRI data including ASD and healthy subjects collected from 17 different sites. We used fMRI data of the same group of subjects which was used in Heinsfeld et al. (2018). This set consists of 505 subjects with ASD and 530 healthy control from all the 17 sites. Table 1 shows the class membership information for each site. ABIDE-I provided the average time series extracted from seven sets of regions of interest (ROIs) based on seven different atlases which are preprocessed using four different pipelines. The data used in our experiments is preprocessed using C-PAC pipeline (Craddock et al., 2013) and is parcellated into 200 functionally homogeneous regions generated using spatially constrained spectral clustering algorithm (Craddock et al., 2012) (CC-200). The preprocessing steps include slice time correction, motion correction, nuisance signal removal, low frequency drifts, and voxel intensity normalization. It is worth mentioning that each site used different parameters and protocols for scanning the data. Parameters like repetition time (TR), echo time (TE), number of voxels, number of volumes, openness or closeness of the eyes while scanning are different among sites.

**Table 1:** Class membership information of ABIDE-I dataset for each individual site.

**Site**	**Caltech**	**CMU**	**KKI**	**Leuven**	**MaxMun**	**NYU**	**OHSU**	**OLIN**	**PITT**	**SBL**	**SDSU**	**Stanford**	**Trinity**	**UCLA**	**UM**	**USM**	**Yale**
ASD	19	14	20	29	24	75	12	19	29	15	14	19	22	54	66	46	28
Healthy control	18	13	28	34	28	100	14	15	27	15	22	20	25	44	74	25	28
Male count	29	21	36	55	48	139	26	29	48	30	29	31	47	86	113	71	40
Female count	8	6	12	8	4	36	0	5	8	0	7	8	0	12	27	0	16
Average age	27	26	10	18	25	15	10	16	18	34	14	9	16	13	14	22	12

### 2.2. ASD-DiagNet: Feature Extraction and Classification

Functional connectivity between brain regions is an important concept in fMRI analysis and is shown to contain discriminatory patterns for fMRI classification. Among correlation measures, Pearson's correlation is mostly used for approximating the functional connectivity in fMRI data (Liang et al., 2012; Baggio et al., 2014; Zhang et al., 2017). It shows the linear relationship between the time series of two different regions. Given two times series, *u* and *v*, each of length *T*, the Pearson's correlation can be computed using the following equation:

(1) $$\begin{array}{l} \rho_{uv}=\frac{\sum_{t=1}^{T}\left(u_{t}-ū\right)\left(v_{t}-\bar{v}\right)}{\sqrt{\sum_{t=1}^{T}{\left(u_{t}-ū\right)}^{2}}\sqrt{\sum_{t=1}^{T}{\left(v_{t}-\bar{v}\right)}^{2}}} \end{array}$$

where ū and $\bar{v}$ are the mean of times series *u* and *v*, respectively. Computing all pairwise correlations results in a correlation matrix $C_{m\times m}$ where *m* is the number of time series (or regions). Due to the symmetric property of Pearson's correlation, we only considered the strictly upper triangle part of the correlation matrix. Since we used CC-200 atlas in which the brain is parcellated into *m* = 200 regions, there are *m* × (*m* − 1)/2 = 19, 900 distinct pairwise Pearson's correlations. In this regard, we selected half of the correlations comprising 1/4 largest and 1/4 smallest values and eliminated the rest. To do so, we first compute the average of correlations among all subjects in training set and then pick the indices of the largest positive and negative values from averaged correlation array. We then pick the correlations at those indices from each sample as our feature vector. Keeping half of the correlations and eliminating the rest reduces the size of input features by a factor of 2. There is no limitation of the number of high- and anti-correlations that should be kept. Removing more features results in higher computational efficiency as well as reducing the chance of overfitting, however removing too many features can also cause losing important patterns.

**Figure 1:**
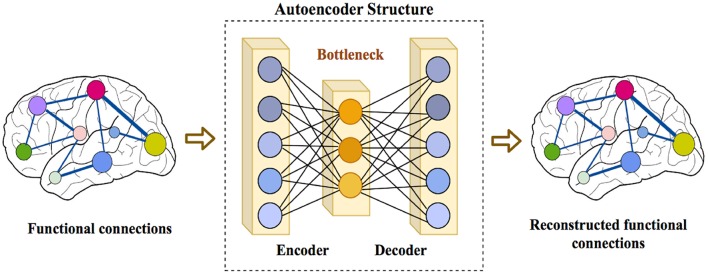
Structure of an autoencoder consisting of an encoder that receives the input data and encodes it into a lower dimensional representation at the bottleneck layer, and a decoder that reconstructs the original input from the bottleneck layer.

In order to further reduce the size of features, we used an autoencoder to extract a lower dimensional feature representation. An autoencoder is a type of feed-forward neural network model, which first encodes its input *x* to a lower dimensional representation,

(2) $$\begin{array}{l} h_{enc}=\phi_{enc}\left(x\right)=\tau \left(W_{enc}x+b_{enc}\right) \end{array}$$

where τ is the hyperbolic tangent activation function (*Tanh*), and *W*_*enc*_ and *b*_*enc*_ represent the weight matrix and the bias for the encoder. Then, the decoder reconstructs the original input data

(3) $$\begin{array}{l} x^{\prime }=\phi_{dec}\left(h_{enc}\right)=W_{dec}h_{enc}+b_{dec} \end{array}$$

where *W*_*dec*_ and *b*_*dec*_ are the weight matrix and bias for the decoder. In this work, we have designed an autoencoder with tied weights, which means $W_{dec}=W_{enc}^{⊤}$. An autoencoder can be trained to minimize its reconstruction error, computed as the Mean Squared Error (MSE) between *x* and its reconstruction, *x*′. The choice of using autoencoder instead of other feature extraction techniques like PCA is its ability to reduce the dimensionality of features in a non-linear way. The structure of an autoencoder is shown in Figure 1.

The lower dimensional data generated during the encoding process contains useful patterns from the original input data with smaller size, and can be used as new features for classification. For the classification task, we used a single layer perceptron (SLP) which uses the bottleneck layer of the autoencoder, *h*_*enc*_, as input, and computes the probability of a sample belonging to the ASD patient class using a sigmoid activation function, σ,

(4) $$\begin{array}{l} f\left(x\right)=\sigma \left(W_{slp}h_{enc}+b_{slp}\right)\\ \;=\sigma \left(W_{slp}\tau \left(W_{enc}x+b_{enc}\right)+b_{slp}\right) \end{array}$$

where *W*_*slp*_ and *b*_*slp*_ are the weight matrix and the bias for the SLP network. The SLP network can be trained by minimizing the Binary Cross Entropy loss, H, using the ground-truth class label, *y*, and the estimated ASD probability for each sample, *f*(*x*):

(5) $$\begin{array}{l} H\left(y,f\left(x\right)\right)=-\left(y\times f\left(x\right)+\left(1-y\right)\times \left(1-f\left(x\right)\right)\right) \end{array}$$

Finally, the predicted class label is determined by thresholding the estimated probability

(6) $$\hat{y}=\{\begin{array}{cc} 1 & \text{if }f(x)\geq 0.5,0,\\ 0 & \text{otherwise}. \end{array}$$

Typically, an autoencoder is fully trained such that its reconstruction error is minimized, then, the features from bottleneck layer, *h*_*enc*_, are used as input for training the SLP classifier, separately. In contrast, here, we train the autoencoder and the SLP classifier simultaneously. This can potentially result in obtaining low dimensional features that have two properties

1. Useful for reconstructing the original data,
2. Contain discriminatory information for the classification task.

This is accomplished by adding the two loss functions, i.e., MSE loss for reconstruction, and Binary Cross Entropy for the classification task, and training both networks jointly. After the joint training process is completed, we further fine-tune the SLP network for a few additional epochs.

### 2.3. Data Augmentation Using Linear Interpolation

Machine learning and especially deep learning techniques can be advantageous if they are provided with enough training data. Insufficient data causes overfitting and non-generalizability of the model (Raschka and Mirjalili, 2017). Large training sets are not always available and collecting new data might be costly like in medical imaging field. In these situations, data augmentation techniques can be used for generating synthetic data using the available training set (Karpathy et al., 2014; Eitel et al., 2015; Wong et al., 2016; Xu et al., 2016; Perez and Wang, 2017). There are a few data augmentation methods proposed for different applications, such as random translation/rotation/cropping (for image data), adding random noise to the features (for general type of data), extracting overlapping windows from the original time series (for time series data), as well as more sophisticated methods such as Generative Adversarial Networks. However, these methods are not either applicable to our data due to the structure of our features, not interpretable, or they may be computationally more intensive than our proposed method.

The data augmentation technique that we propose in this study is inspired by Synthetic Minority Over-sampling Technique (SMOTE) (Chawla et al., 2002). SMOTE is an effective model which is used for oversampling the data in minority class of imbalanced datasets. SMOTE generates synthetic data in feature space by using the nearest neighbors of a sample. After k-nearest neighbors of sample *p* are found ({*q*_1_, *q*_2_, ..., *q*_*k*_}), a random neighbor is selected (*q*_*r*_) and the synthetic feature vector is computed using the following equation:

(7) $$\begin{array}{l} p^{\prime }=\alpha \times p+\left(1-\alpha \right)\times q_{r} \end{array}$$

In this equation, α is a random number selected uniformly in the range [0, 1]. Finding the nearest neighbors of a sample is based on a distance or similarity metric. In our work, the samples have feature vectors of size 9, 950 (half of the correlations). One idea for computing nearest neighbors is to use Euclidean distance, however, computing the pairwise Euclidean distances with 9, 950 features is not efficient. In order to compute the similarity between samples and finding the nearest neighbors, we used a measure called Extended Frobenius Norm (EROS). This measure computes the similarity between two multivariate time series (MTS) (Yang and Shahabi, 2004). fMRI data consists of several regions each having a time series so we can consider it as a multivariate time series. Our previous study on ADHD disorder has shown that EROS is an effective similarity measure for fMRI data and using it along with k-Nearest-Neighbor achieves high classification accuracy (Eslami and Saeed, 2018b). This motivated us to utilize it as part of the data augmentation process. EROS computes the similarity between two MTS items *A* and *B* based on eigenvalues and eigenvectors of their covariance matrices using the following equation:

(8) $$\begin{array}{l} EROS\left(A,B,w\right)=\overset{n}{\underset{i=1}{\sum }}w_{i}|\langle a_{i},b_{i}\rangle |\\ \;=\overset{n}{\underset{i=1}{\sum }}w_{i}|cos\theta_{i}| \end{array}$$

where, θ_*i*_ is the cosine of the angle between *i*_*th*_ corresponding eigenvectors of covariance matrices of multivariate time series *A* and *B*. Furthermore, *w* is the weight vector which is computed based on eigenvalues of all MTS items using Algorithm 1. This algorithm computes the weight vector *w* by normalizing eigenvalues of each MTS item followed by applying an aggregate function *f* (here, we used mean) to all eigenvalues over the entire training dataset and finally normalizing them so that $\overset{n}{\underset{i=1}{\sum }}w_{i}=1$.

**Table:** **Algorithm 1**: Computing weight vector for EROS (Yang and Shahabi, 2004)

**Input:** An *n* × *N* matrix *S*, where *n* is the number of variables for the dataset and *N* is the number of MTS items in the dataset.
Each column vector *s*_*i*_ in *S* represents all the eigenvalues for *i*_*th*_ MTS item in the dataset. *s*_*ij*_ is a value at column *i* and row *j* in *S*. s_*i_ is *i*_*th*_ row in *S*. *s*_i*_ is *i*_*th*_ column
1: for *i* = 1 to *N* **do**
2: $s_{i}\;\leftarrow \;s_{i}/\;\overset{n}{\underset{j=1}{\sum }}s_{ij}$
3: **end for**
4: **for** *i* = 1 to *n* **do**
5: *w*_*i*_ ← *f*(s_*i_)
6: **end for**
7: **for** *i* = 1 to *n* **do**
8: $w_{i}\;\leftarrow \;w_{i}/\overset{n}{\underset{j=1}{\sum }}w_{j}$
9: **end for**

**Table:** **Algorithm 2**: Data augmentation using EROS similarity measure

**Input:** Training dataset of size N
1: **for** *i* = 1 to *N* **do**
2: Find 5 nearest neighbors to *i* using EROS
3: *j* ← A random sample among nearest neighbors
4: *r* ← Random number in the range [0, 1]
5: $x_{i+N}^{*}\;\leftarrow \;\alpha \times x_{i}+\left(1-\alpha \right)\times x_{j}$
6: **end for**

The dimension of each sample's covariance matrix is *m* × *m*, where *m* is the number of brain regions. The covariance matrix of each subject is pre-computed in the beginning and is re-used when the sample is selected as a candidate. In order to further reduce the time needed for computing the pairwise similarities, we considered using the first two eigenvectors of each sample. Our experiments showed that this simplification does not affect the results while reducing the running time significantly compared to using all eigenvectors and eigenvalues.

**Figure 2:**
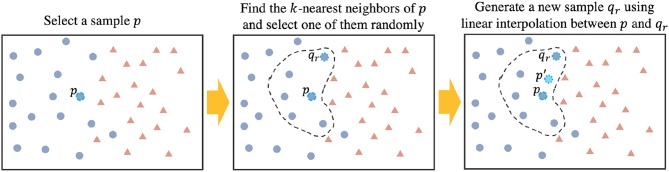
Generating new artificial data: Step (1) Selecting a sample (*p*). Step (2) Find *k*-nearest neighbors of *p* from the same class, and pick one random neighbor (*q*_*r*_). Step (3) Generate new sample *p*′ using *p* and *q*_*r*_ by linear interpolation.

**Figure 3:**
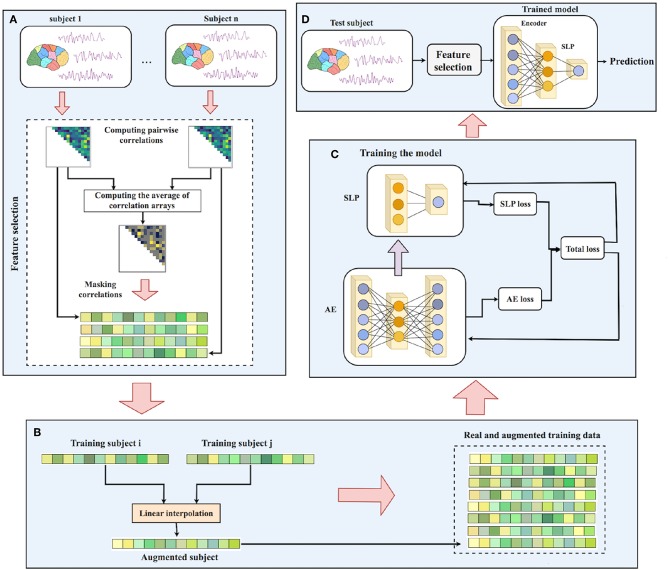
Workflow of ASD-DiagNet: **(A)** Pairwise Pearson's correlations for each subject in the training set is computed. The average of all correlation arrays is computed and the position of 1/4 largest and 1/4 smallest values in the average array is considered as a mask. Masked correlation array of each sample is considered as its feature vectors. **(B)** A set of artificial samples is generated using the feature vectors of training samples. **(C)** Autoencoder and SLP are jointly trained by adding up their training loss in each iteration. **(D)** For a test subject, the features are extracted using the mask generated in part A, followed by passing the features through the encoder part of the autoencoder, and finally predicting its label using the trained SLP.

Now, using EROS as the similarity measure, our data augmentation process is shown in Algorithm 2. After finding *k* = 5 nearest neighbors of each sample *i* in the training set, one of them is randomly selected, a new sample is generated using linear interpolation between the selected neighbor and sample *i*. Choosing *k* = 5 was based on the original implementation of SMOTE algorithm (Chawla et al., 2002). Our experiments did not show a significant change in the results when using different values of *k*. Using this approach, one synthetic sample is created for each training point which results in doubling the size of the training set. Figure 2 shows the data augmentation process and Figure 3 shows the overall process of ASD-DiagNet method.

## 3. Experiments and Results

For all the experiments reported in this section, we used a Linux server running Ubuntu Operating System. The server contains two Intel Xeon E5-2620 Processors at 2.40 GHz with a total 48 GBs of RAM. The system contains an NVIDIA Tesla K-40c GPU with 2, 880 CUDA cores and 12 GBs of RAM. CUDA version 8 and PyTorch library were used for conducting the experiments.

We evaluated ASD-DiagNet model in two phases by performing *k*-fold cross validation. In the first phase, the model was evaluated using the whole 1, 035 subjects from all sites and in the second phase, the model was evaluated for each site separately. As stated earlier, data centers may have used different experimental parameters for scanning fMRI images, so considering all of them in the same pool determines how our model generalizes to data with heterogeneous scanning parameters. On the other hand, by considering each data center separately, fewer subjects are available for training the model and the results indicate how it performs on small datasets. In each of these experiments, the effect of data augmentation is evaluated.

The value of *k* in *k*-fold cross validation must be chosen such that train/test partitions are representative of the whole dataset. Since the whole dataset contains a lot more samples than each individual site, using a large value of *k* like 10 in *k*-fold cross validation provides more samples in the training process. This helps the model to capture more information from the data while leaving enough test samples to measure the ability of the model in classifying unseen data. On the other hand, we are dealing with a small number of samples in some of the sites, for example, CMU which only contains 27 samples. Hence performing *k*-fold cross validation with large values of *k* like 10 results in only 2–3 samples in test set and increases the variance of cross-validation estimation, so we chose *k* = 5 when analyzing each site separately. Other studies such as Heinsfeld et al. (2018) used the same values of *k* for performing *k*-fold cross validation.

We report accuracy, sensitivity, and specificity of different methods for evaluating their classification performance. Accuracy measures the proportion of correctly classified subjects (actual ASD classified as ASD and actual healthy classified as healthy). Sensitivity represents the proportion of actual ASD subjects which are correctly classified as ASD and specificity measures the proportion of actual healthy subjects which are classified as healthy. We also compared the performance of each model's diagnostic test by their Receiver Operating Characteristic (ROC) curves. The area under ROC curves (AUC) shows the capability of the model for distinguishing between ASD and healthy subjects based on different thresholds. The higher AUC value indicates that the model is better in distinguishing between ASD and healthy subjects. We compared the performance of ASD-DiagNet with three other baselines: SVM, random forest and the method proposed by Heinsfeld et al. (2018). Hyperparameter tuning for SVM and random forest classifiers are performed by grid search technique. Hyperparameters such as kernel type, regularization constant (C), kernel coefficient (γ) for SVM, and the number of trees as well as the function to measure the quality of a split for random forest are tuned using grid search. SVM and random forest were trained using 19, 900 pairwise Pearson's correlations for each subject. The implementations of the grid search, SVM, and random forest are carried out using the built-in functions provided by scikit-learn library. In order to speed up the grid search, it is parallelized on 10 cores.

The following subsections explain each experiment in more details.

### 3.1. Phase 1: Experiments Using the Whole Dataset

In this phase, we performed 10-fold cross-validation on the whole 1, 035 subjects using CC-200 atlas. Table 2 compares accuracy, sensitivity, and specificity of our approach with Heinsfeld et al. (2018), random forest, and SVM. As the results show, ASD-DiagNet achieves 70.3% which outperforms other methods.^1^

**Table 2:** Classification performance using 10-fold cross-validation on the whole dataset; Note that our proposed approach, ASD-DiagNet (with data augmentation) achieves the highest accuracy among other methods.

**Method**	**Accuracy**	**Sensitivity**	**Specificity**
ASD-DiagNet	**70.3**	68.3	72.2
ASD-DiagNet (no aug.)	69.4	69.6	69.2
SVM	68.3	64.4	72
Random forest	66.3	60.8	71.4
Heinsfeld et al., 2018	65.4	61	69.3

*Bold values show the highest accuracy among all methods*.

**Figure 4:**
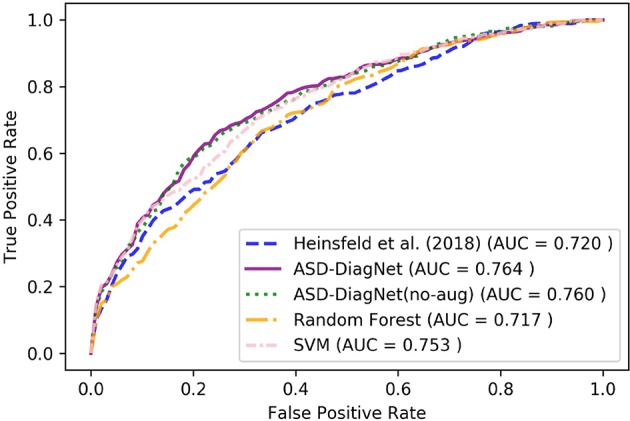
ROC curves of different methods for classification of whole dataset using CC-200 parcellation.

The proposed data augmentation helps to improve the results by around 1%. Based on Figure 4, ASD-DiagNet (with and without data augmentation) achieved higher area under comparing to other methods.

### 3.2. Phase 2: Intra-Site Evaluation

In this phase, we performed 5-fold cross-validation on each site separately using CC-200 atlas. The accuracy of each method is provided in Table 3. Based on these results, our method achieves the highest accuracy in most cases (10 out of 17 sites) and outperforms other methods on average. In addition, note that the proposed data augmentation helps improving the result around 3% overall. Especially, for OHSU, the data augmentation improves the accuracy significantly (10% increase). However, in a couple of datasets no improvement is observed (e.g., MaxMun). These datasets have shown low prediction accuracy by other methods as well. In these cases, the artificial data generated by data augmentation does not improve the results since the functional connectivity of the original data does not carry enough discriminatory information that can be used by the classifiers.

**Table 3:** Classification accuracy using 5-fold cross-validation on individual data centers using our proposed method, ASD-DiagNet (with and without data augmentation), compared with other methods.

**Site**	**ASD-DiagNet**	**ASD-DiagNet (no aug.)**	**Heinsfeld et al., 2018**	**SVM**	**Random- Forest**
Caltech	52.8	49.9	52.3	46.9	**54.2**
CMU	**68.5**	67.4	45.3	66.6	62.4
KKI	**69.5**	68.6	58.2	66.4	66.6
Leuven	**61.3**	57	51.8	59.8	59.8
MaxMun	48.6	51.4	**54.3**	53.8	49.2
NYU	68	65.1	64.5	**71.4**	61.8
**OHSU**	82	71.9	74	79.4	54.3
Olin	**65.1**	58.8	44	59.5	52.2
Pitt	**67.8**	65.9	59.8	66.3	59.9
SBL	51.6	47.5	46.6	**60**	48.3
SDSU	63	61.3	**63.6**	58.7	62.7
Stanford	**64.2**	53	48.5	51.4	62.1
Trinity	54.1	51.2	**61**	53.1	54.5
UCLA	**73.2**	70.3	57.7	72.1	69.3
USM	68.2	65.1	62	**73.2**	58
UM	63.8	**65.7**	57.6	64.2	64.8
Yale	**63.6**	61.7	53	61.6	55.3
Average	**63.8**	60.7	56.1	62.6	58.6

*Bold and color values corresponds to highest accuracy achieved among all datasets*.

### 3.3. Running Time

We measured the running time of performing 10-fold cross validation by different approaches. The training and evaluation for all methods are performed on the same Linux system (described in section 3). The running time needed by each method is as follows: 41 min by ASD-DiagNet, 20 min by ASD-DiagNet (no aug.), 7 h and 48 min by SVM, 17 min by random forest and 6 h by Heinsfeld et al. (2018). As can be observed, ASD-DiagNet performs significantly faster than SVM and Heinsfeld et al. (2018). The data augmentation doubles the size of the training set by generating one artificial sample per subject in the training set. As a result, the data augmentation increases the computation time by a factor of 2.

### 3.4. Experiment on Other Parcellations

We tested ASD-DiagNet on two other ROI atlases besides CC-200: Automated Anatomical Labeling (AAL) and Talaraich and Tournoux (TT) which parcellate the brain into 116 and 97 regions respectively. The data for these parcellations is provided by ABIDE-I consortium. Similar to CC-200 atlas, for each parcellation, half of the correlations (keeping the 1/4 largest and 1/4 smallest values, and removing the rest intermediate values) are selected as input features to the model. The resulting average accuracy, sensitivity, and specificity of performing 10-fold cross-validation on the whole dataset using different approaches for AAL and TT are shown in Tables 4, 5.

**Table 4:** Classification accuracy using 10-fold cross-validation on the whole dataset based on AAL atlas.

**Method**	**Accuracy**	**Sensitivity**	**Specificity**
ASD-DiagNet	**67.5**	63.4	71.5
ASD-DiagNet (no aug.)	64.5	60.9	68
SVM	**67.5**	63.9	70.9
Random forest	65	56.8	72.7
Heinsfeld et al., 2018	63.3	58.6	67.8

*Bold values show the highest accuracy among all methods*.

**Table 5:** Classification accuracy using 10-fold cross-validation on the whole dataset based on TT atlas.

**Method**	**Accuracy**	**Sensitivity**	**Specificity**
ASD-DiagNet	65.3	63.4	66.9
ASD-DiagNet (no aug.)	65.2	61.1	69
SVM	**66.4**	61.6	71
Random forest	65.1	60.3	69.7
Heinsfeld et al., 2018	63.2	59.8	66.4

*Bold values show the highest accuracy among all methods*.

**Figure 5:**
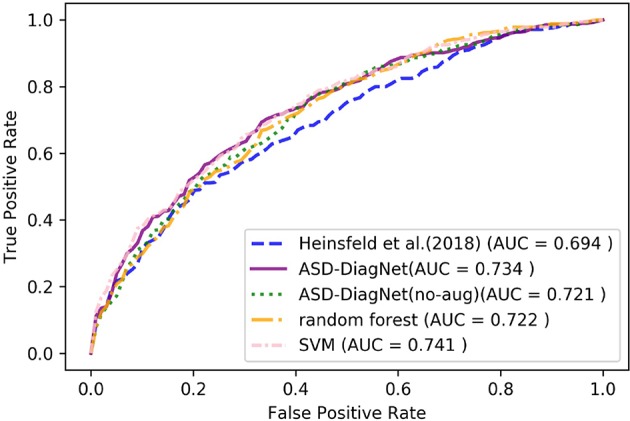
ROC curves of different methods for classification of whole dataset using AAL parcellation.

**Figure 6:**
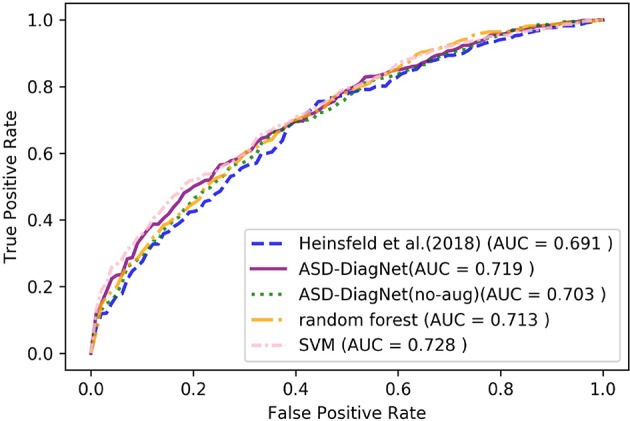
ROC curves of different methods for classification of whole dataset using TT parcellation.

For AAL parcellation, ASD-DiagNet and SVM outperform other techniques with the classification accuracy of 67.5% and achieve competitive result for TT atlas. Note that the classification accuracy obtained using these parcellations are below the accuracy obtained using CC-200 atlas, which implies that the pairwise correlations among CC-200 regions contain more discriminatory patterns than AAL and TT atlases. Based on Figures 5, 6, SVM and ASD-DiagNet achieved higher AUC than other methods.

### 3.5. Experiments on Young Age Group

**Figure 7:**
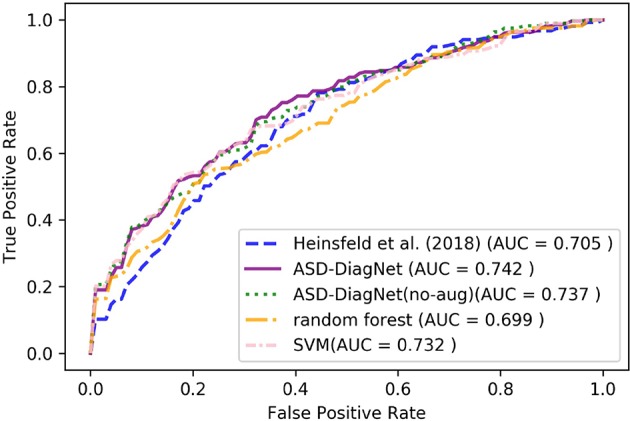
ROC curves of different methods for classification of subjects below the age of 15 using CC-200 parcellation.

Diagnosing ASD at early ages and starting medical treatment can have a positive effect on the patient's life. In this experiment, we evaluated our proposed method as well as other baselines on subjects below the age of 15 (550 subjects in ABIDE dataset containing 448 males and 102 females) using CC-200 atlas. Considering this subset of subjects, the classification performance, as well as ROC curves of performing 10-fold cross-validation of different methods are provided in Table 6 and Figure 7.

**Table 6:** Classification accuracy using 10-fold cross-validation on the subjects below the age of 15.

**Method**	**Accuracy**	**Sensitivity**	**Specificity**
ASD-DiagNet	**68.2**	66.7	69.4
ASD-DiagNet (no aug.)	66.9	59.2	74.3
SVM	66.9	64.5	69.2
Random forest	64.3	57.4	70.8
Heinsfeld et al., 2018	65.2	62.1	68.3

As can be observed from the results, ASD-DiagNet achieves higher accuracy as well as higher AUC value compared to other methods. The overall accuracy is around 2% below the accuracy achieved for classification of the whole dataset, which we believe is due to the smaller training set.

## 4. Conclusion and Future Work

In this paper, we targeted the problem of classifying subjects with ASD disorder from healthy subjects. We used fMRI data provided by ABIDE consortium, which has been collected from different brain imaging centers. Our approach, called *ASD-DiagNet*, is based on using the most correlated and anti-correlated connections of the brain as feature vectors and using an autoencoder to extract lower dimensional patterns from them. The autoencoder and a SLP are trained in a joint approach for performing feature selection and classification. We also proposed a data augmentation method in order to increase the number of samples using the available training set. We tested this method by performing 10-fold cross-validation on the whole dataset and achieved 70.3% accuracy in 40 min. The running time of our approach is significantly shorter than 6 h needed by the state of the art method while achieving higher classification accuracy. In another experiment, we evaluated our method by performing 5-fold cross-validation on each data center, separately. The average result shows significant improvement in accuracy compared to the state of the art method. In this case, data augmentation helps to improve the accuracy by around 3%. A different range of accuracies can be observed among sites, from low accuracies in sites such as Caltech and MaxMun to higher accuracies for OHSU and UCLA. The variable accuracy among different sites can also be observed in other studies (Nielsen et al., 2013; Heinsfeld et al., 2018). It should be noted that the protocols and parameters used for scanning the subjects are heterogeneous among sites, which can cause variability in the functional patterns among different subjects. Also, the difference in demographic information among the datasets, such as age, IQ, and gender, makes the data distribution different among them. These differences could be the reason for variable accuracies. We will consider this issue in our future works by involving the demographic information of the samples in data augmentation and the learning process. This will help the classifier to learn associations between functional connectivity patterns and demographic features which decreases the disparity among accuracies of different sites. We will also analyze other parcellations such as Power-264 by Power et al. (2011). The functional network constructed using this parcellation has shown promising results in diagnosing brain disorders (Greene et al., 2016; Khazaee et al., 2016).

Overall, experiments on different parcellations as well as subjects below the age of 15 show higher accuracy and AUC value for ASD-DiagNet comparing to other methods. These results demonstrate that our approach can be used for both intra-site brain imaging data, which are usually small sets generated in research centers, and bigger multi-site datasets like ABIDE in a reasonable amount of time.

While our model has shown promising results for diagnosing ASD disorder, there is still room for improvement by fusing structural and phenotypic information of the subjects to the functional patterns and creating hybrid features. Combination of discriminatory information provided by these three sources could increase the prediction accuracy of ASD. We consider this feature fusion as one of the future directions of our study. Another direction that we will pursue is improving the data augmentation strategy. Overall, the proposed data augmentation has improved the accuracy by generating synthetic data, but in a couple of cases low or no improvement is observed. Optimizing the current data augmentation method and considering the structural and phenotypic data for generating new samples could potentially improve the data augmentation process, and as a result, may lead to increase the diagnosis accuracy.

## Data Availability Statement

The datasets analyzed for this study can be found in the ABIDE-I repository (Craddock et al., 2013).

## Author Contributions

TE, VM, and FS conceived the study. TE pursued the implementation of the method, conducted the experiments and generated the results. TE and FS wrote the manuscript. AL and AF provided critical feedback and suggestions for performing the experiments. FS, AF, and VM provided valuable suggestions in writing the manuscript.

### Conflict of Interest

The authors declare that the research was conducted in the absence of any commercial or financial relationships that could be construed as a potential conflict of interest.

## Footnotes

^1^We like to mention that Heinsfeld et al. (2018) reported 70% accuracy in their paper, however, the accuracy we reported here is the result of running their method on our system using their default parameters and the code they provided online. The different results observed here could be due to some missing details in the implementation.
